# Supplementary material for: Krypton oxides under pressure
Source: Sci Rep. 2016 Feb 2;6:18938. doi: 10.1038/srep18938 (PMC4735652; doi:10.1038/srep18938)
Supplement: Supplementary Information [file srep18938-s1.doc]

**Krypton oxides under pressure**

**(SUPLEMENTARY MATERIAL)**

Patryk Zaleski-Ejgierd ^a^,* and Paweł Łata ^a^,**^+^**

^a^ Institute of Physical Chemistry, ul. M. Kasprzaka 44/52, 01-224 Warsaw, Poland

*pze.work@gmail.com, **^+^**plata@ichf.edu.pl

**
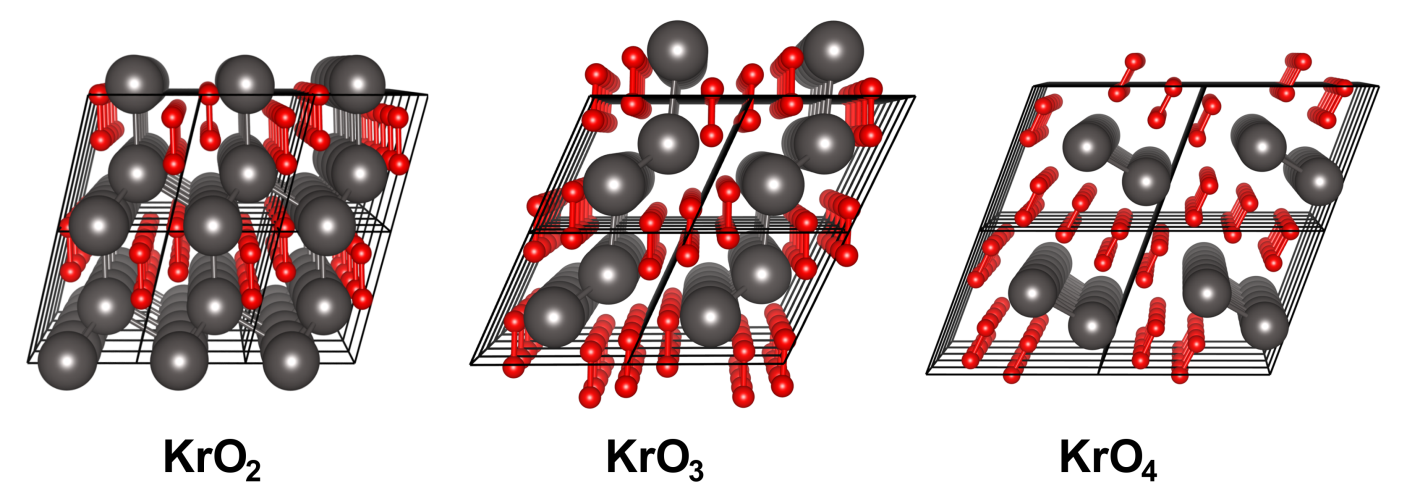
**

**Figure S1:** Schematic representation of the selected most-stable phases of KrO_2_, KrO_3_ and KrO_4_ and their corresponding unit cells at 300 GPa (large gray spheres – krypton; small red spheres – oxygen). For illustration purposes, and clarity, selected Kr-Kr and O-O contacts are depicted (Kr-Kr < 3.0 Å and O-O < 1.6 Å). Note the lack of short Kr-O bonds (Kr-O < 1.9 Å) and the increasing level of segregation with the increasing oxygen content.

**Computational details:**

The search for stable structures of KrO and KrO_n_ (n = 2-4) was conducted with the application of the USPEX evolutionary algorithm. Calculations were performed at 100, 200, 300, 400 and 500 GPa with system sizes of 1, 2, and 4 formula units per cell (Z). For each computational point (stoichiometry/pressure/Z) one hundred random structures were generated in the first generation. Each subsequent generation consisted of 30% new random structures (random + softmutation) and 70% offspring structures. At least 15 generations were calculated for each computational point. A total number of approximately 80.000-100.000 structures have been generated and analyzed (including, of course, duplicates).

USPEX input example (KrO, Z=1):

--------------------------------------------------------------------------------------------------------------------------------------------------------

PARAMETERS EVOLUTIONARY ALGORITHM

******************************************

******************************************

* TYPE OF RUN AND SYSTEM *

******************************************

******************************************

USPEX : calculationMethod (USPEX, VCNEB, META)

300 : calculationType (dimension: 0-3; molecule: 0/1; varcomp: 0/1)

1 : optType (1=enthalpy, 2=volume, 3=hardness, 4=struc_order, 5=aver_dist)

%%%%%%%%%%%%%%%%%%%%%%%%%%%%%%%%%%%%%%%%%%%%%%%

% What symmetry(s) have to be satisfied by the randomly created structures

% symmetries

2-230

% endSymmetries

% numbers of specices (ions/molecules/blocks) of each type

% numSpecices

1 1

% EndNumSpecices

%%%%%%%%%%%%%%%%%%%%

% Here come the atomic numbers of the atoms involved

% atomType

36 8

% EndAtomType

%%%%%%%%%%%%%%%%%%%%

% valences

4 2

% endValences

******************************************

* POPULATION *

******************************************

100 : populationSize (how many individuals per generation)

100 : initialPopSize

20 : numGenerations (how many generations shall be calculated)

15 : stopCrit

******************************************

* SURVIVAL OF THE FITTEST AND SELECTION *

******************************************

0 : reoptOld

0.6 : bestFrac

******************************************

******************************************

* VARIATION OPERATORS *

******************************************

******************************************

0.70 : fracGene (fraction of generation produced by heredity)

0.15 : fracRand (fraction of generation produced randomly from space groups)

0.15 : fracAtomsMut (fraction of the generation produced by softmutation)

% structures produced by lattice mutation = 1.0-(FracGene+FracPerm+FracRotMut)

% so don't need to specify explicitly

0.50 : mutationRate (standard deviation of the epsilons in the strain matrix)

1.00 : DisplaceInLatmutation

****************************************

* CONSTRAINTS *

****************************************

2.0 : minVectorLength ( minimal length of any lattice vector)

%%%%%%%%%%%%%%%%

% IonDistances

0.5 1.0

1.0 1.5

% EndDistances

%%%%%%%%%%%%%%%%

*****************************************

* CELL *

*****************************************

% The following is what you know about the lattice. If you know the lattice

% vectors, type them in as 3x3 matrix. If not, type the estimated volume.

% For variable composition - type the estimated atomic volume for each element.

% Latticevalues (this word MUST stay here, type values below)

50.0

% Endvalues (this word MUST stay here)

*****************************************

* DETAILS OF AB INITIO CALCULATIONS *

*****************************************

% supported: 1-vasp, 2-siesta, 3-gulp, 4-LAMMPS, 5-NeuralNetworks

% 6-dmacrys, 7-cp2k, 8-QuantumEspresso, 9-ASE, 10-ATK, 11-CASTEP

abinitioCode (which code from CommandExecutable shall be used for calculation? )

1 1 1 1 1

ENDabinit

%Resolution for KPOINTS - one number per step or just one number in total)

% KresolStart

0.15 0.12 0.09 0.08 0.07

% Kresolend

% commandExecutable

/home/pablo/uspex/vasp > log

% EndExecutable

10 : numParallelCalcs (how many parallel calculations shall be performed)

1 : whichCluster (0: no-job-script, 1: local submission, 2: remote submission)

0.010 : toleranceFing (tolerance for identical structures)

--------------------------------------------------------------------------------------------------------------------------------------------------------

**Table S1.** Selected number of Kr-Kr, Kr-O and O-O contacts and the corresponding contact lengths [Å], calculated at 300 GPa for selected enthalpically preferred phases of KrO.

| **Structure** | **Kr-Kr**  **[Å]** | **Kr-O**  **[Å]** | **O-O**  **[Å]** | **Volume**  **[Å^3^/f.u.]** | **No. of contacts**  **(with Kr-O < 2.0 Å)**  **\** |
| --- | --- | --- | --- | --- | --- |
| Phase A | 2.455 (x4)  2.556 (x2)  3.073 (x2) | 1.863 (x2)  2.126 (x2)  2.301 (x4) | 2.455 (x4) | 14.90 | 2 |
| Phase B | 2.495 (x2)  2.500 (x1)  2.602 (x2)  2.740 (x1) | 2.119 (x2)  2.158 (x1) | 1.235 (x1) | 15.27 | 0 |
| Phase C | 2.455 (x6) | 2.126 (x8) | 2.455 (x6) | 14.79 | 0 |
| Phase D | 2.513 (x4)  2.427 (x2) | 1.807 (x2)  2.185 (x2)  2.314 (x4) | 2.244 (x2) | 14.99 | 2 |

**Structures (all data from 300 GPa)**

**KrO, Phase A (C2/m, Z=1)**

data_PhaseI-[C2overm]-0.001-prim-300GPa

_audit_creation_date 2015-06-17

_audit_creation_method ''

_symmetry_space_group_name_H-M ' '

_symmetry_Int_Tables_number 0

_symmetry_cell_setting monoclinic

loop_

_symmetry_equiv_pos_as_xyz

x,y,z

-y,-x,-z

-x,-y,-z

y,x,z

_cell_length_a 2.4545

_cell_length_b 2.4545

_cell_length_c 3.7254

_cell_angle_alpha 117.3313

_cell_angle_beta 117.3313

_cell_angle_gamma 102.4743

loop_

_atom_site_label

_atom_site_type_symbol

_atom_site_fract_x

_atom_site_fract_y

_atom_site_fract_z

_atom_site_U_iso_or_equiv

_atom_site_adp_type

_atom_site_occupancy

Kr1 Kr 0.00000 0.00000 0.00000 0.01267 Uiso 1.00

O1 O -0.00000 -0.00000 0.50000 0.01267 Uiso 1.00

**KrO, Phase B (C2/m, Z=2)**

data_PhaseI-[C2overm]-0.001-prim-300GPa

_audit_creation_date 2015-06-17

_audit_creation_method ''

_symmetry_space_group_name_H-M ' '

_symmetry_Int_Tables_number 0

_symmetry_cell_setting monoclinic

loop_

_symmetry_equiv_pos_as_xyz

x,y,z

-y,-x,-z

-x,-y,-z

y,x,z

_cell_length_a 2.4545

_cell_length_b 2.4545

_cell_length_c 3.7254

_cell_angle_alpha 117.3313

_cell_angle_beta 117.3313

_cell_angle_gamma 102.4743

loop_

_atom_site_label

_atom_site_type_symbol

_atom_site_fract_x

_atom_site_fract_y

_atom_site_fract_z

_atom_site_U_iso_or_equiv

_atom_site_adp_type

_atom_site_occupancy

Kr1 Kr 0.00000 0.00000 0.00000 0.01267 Uiso 1.00

O1 O -0.00000 -0.00000 0.50000 0.01267 Uiso 1.00

**KrO, Phase C (Pm3̅m, Z=2)**

data_PhaseIII-[Pm-3m]-0.001-prim-300GPa

_audit_creation_date 2015-06-17

_audit_creation_method ''

_symmetry_space_group_name_H-M 'PM-3M'

_symmetry_Int_Tables_number 221

_symmetry_cell_setting cubic

loop_

_symmetry_equiv_pos_as_xyz

x,y,z

-x,-y,z

-x,y,-z

x,-y,-z

z,x,y

z,-x,-y

-z,-x,y

-z,x,-y

y,z,x

-y,z,-x

y,-z,-x

-y,-z,x

y,x,-z

-y,-x,-z

y,-x,z

-y,x,z

x,z,-y

-x,z,y

-x,-z,-y

x,-z,y

z,y,-x

z,-y,x

-z,y,x

-z,-y,-x

-x,-y,-z

x,y,-z

x,-y,z

-x,y,z

-z,-x,-y

-z,x,y

z,x,-y

z,-x,y

-y,-z,-x

y,-z,x

-y,z,x

y,z,-x

-y,-x,z

y,x,z

-y,x,-z

y,-x,-z

-x,-z,y

x,-z,-y

x,z,y

-x,z,-y

-z,-y,x

-z,y,-x

z,-y,-x

z,y,x

_cell_length_a 2.4548

_cell_length_b 2.4548

_cell_length_c 2.4548

_cell_angle_alpha 90.0000

_cell_angle_beta 90.0000

_cell_angle_gamma 90.0000

loop_

_atom_site_label

_atom_site_type_symbol

_atom_site_fract_x

_atom_site_fract_y

_atom_site_fract_z

_atom_site_U_iso_or_equiv

_atom_site_adp_type

_atom_site_occupancy

Kr1 Kr 0.00000 0.00000 0.00000 0.01267 Uiso 1.00

O1 O 0.50000 0.50000 0.50000 0.01267 Uiso 1.00

**KrO, Phase D (Imma, Z=2)**

data_PhaseV-[IMMA]-0.001-prim-300GPa

_audit_creation_date 2015-06-17

_audit_creation_method ''

_symmetry_space_group_name_H-M ' '

_symmetry_Int_Tables_number 0

_symmetry_cell_setting orthorhombic

loop_

_symmetry_equiv_pos_as_xyz

x,y,z

y-z+1/2,x-z,-z+1/2

-y+z+1/2,-y,x-y+1/2

-x,-x+z,-x+y

-x,-y,-z

-y+z+1/2,-x+z,z+1/2

y-z+1/2,y,-x+y+1/2

x,x-z,x-y

_cell_length_a 3.4940

_cell_length_b 3.4940

_cell_length_c 3.4940

_cell_angle_alpha 92.0049

_cell_angle_beta 111.9075

_cell_angle_gamma 126.2924

loop_

_atom_site_label

_atom_site_type_symbol

_atom_site_fract_x

_atom_site_fract_y

_atom_site_fract_z

_atom_site_U_iso_or_equiv

_atom_site_adp_type

_atom_site_occupancy

Kr1 Kr 0.50000 1.00000 1.00000 0.01267 Uiso 1.00

O1 O 0.07565 0.32565 0.75000 0.01267 Uiso 1.00

**KrO_2_**

data_VESTA_phase_1

_pd_phase_name 'Ö'

_cell_length_a 3.50420

_cell_length_b 4.17872

_cell_length_c 3.50016

_cell_angle_alpha 114.78815

_cell_angle_beta 60.00959

_cell_angle_gamma 114.83594

_symmetry_space_group_name_H-M 'P 1'

_symmetry_Int_Tables_number 1

loop_

_symmetry_equiv_pos_as_xyz

'x, y, z'

loop_

_atom_site_label

_atom_site_occupancy

_atom_site_fract_x

_atom_site_fract_y

_atom_site_fract_z

_atom_site_adp_type

_atom_site_B_iso_or_equiv

_atom_site_type_symbol

Kr1 1.0 0.404216 0.420461 0.224569 Biso 1.000000 Kr

Kr2 1.0 0.621574 0.071234 0.440803 Biso 1.000000 Kr

O1 1.0 0.209625 0.835265 0.028743 Biso 1.000000 O

O2 1.0 0.816166 0.656452 0.636641 Biso 1.000000 O

O3 1.0 0.928540 0.991208 0.747023 Biso 1.000000 O

O4 1.0 0.097266 0.500502 0.918349 Biso 1.000000 O

**KrO_3_**

data_VESTA_phase_2

_pd_phase_name 'º'

_cell_length_a 3.44288

_cell_length_b 4.04067

_cell_length_c 4.10831

_cell_angle_alpha 111.31139

_cell_angle_beta 112.96554

_cell_angle_gamma 93.44381

_symmetry_space_group_name_H-M 'P 1'

_symmetry_Int_Tables_number 1

loop_

_symmetry_equiv_pos_as_xyz

'x, y, z'

loop_

_atom_site_label

_atom_site_occupancy

_atom_site_fract_x

_atom_site_fract_y

_atom_site_fract_z

_atom_site_adp_type

_atom_site_B_iso_or_equiv

_atom_site_type_symbol

Kr1 1.0 0.895710 0.391661 0.651654 Biso 1.000000 Kr

Kr2 1.0 0.289738 0.607954 0.350719 Biso 1.000000 Kr

O1 1.0 0.213051 0.057992 0.173249 Biso 1.000000 O

O2 1.0 0.972397 0.941626 0.829127 Biso 1.000000 O

O3 1.0 0.631399 0.271153 0.058296 Biso 1.000000 O

O4 1.0 0.554057 0.728465 0.944071 Biso 1.000000 O

O5 1.0 0.795293 0.859081 0.288746 Biso 1.000000 O

O6 1.0 0.390151 0.140535 0.713626 Biso 1.000000 O

**KrO_4_**

data_VESTA_phase_3

_pd_phase_name 'Ö'

_cell_length_a 5.61010

_cell_length_b 5.01319

_cell_length_c 2.19519

_cell_angle_alpha 90.00022

_cell_angle_beta 90.00017

_cell_angle_gamma 69.44497

_symmetry_space_group_name_H-M 'P 1'

_symmetry_Int_Tables_number 1

loop_

_symmetry_equiv_pos_as_xyz

'x, y, z'

loop_

_atom_site_label

_atom_site_occupancy

_atom_site_fract_x

_atom_site_fract_y

_atom_site_fract_z

_atom_site_adp_type

_atom_site_B_iso_or_equiv

_atom_site_type_symbol

Kr1 1.0 0.447862 0.587224 0.506148 Biso 1.000000 Kr

Kr2 1.0 0.861214 0.340656 0.006154 Biso 1.000000 Kr

O1 1.0 0.068485 0.625606 0.006216 Biso 1.000000 O

O2 1.0 0.240575 0.302282 0.506222 Biso 1.000000 O

O3 1.0 0.815371 0.983587 0.506212 Biso 1.000000 O

O4 1.0 0.522656 0.166993 0.006217 Biso 1.000000 O

O5 1.0 0.087205 0.853028 0.006215 Biso 1.000000 O

O6 1.0 0.221882 0.074849 0.506218 Biso 1.000000 O

O7 1.0 0.786414 0.760900 0.506209 Biso 1.000000 O

O8 1.0 0.493694 0.944307 0.006214 Biso 1.000000 O
